# Clinical features in antiglycine receptor antibody-related disease: a case report and update literature review

**DOI:** 10.3389/fimmu.2024.1387591

**Published:** 2024-06-17

**Authors:** Xiaoke Wu, Haifeng Zhang, Mengmeng Shi, Shaokuan Fang

**Affiliations:** ^1^ Department of Neurology, Neuroscience Centre, The First Hospital of Jilin University, Changchun, China; ^2^ Department of Neurology, The First Affiliated Hospital of Zhengzhou University, Zhengzhou, China

**Keywords:** antiglycine receptor antibody, progressive encephalomyelitis with rigidity and myoclonus, stiff-person syndrome, epilepsy, autoimmune

## Abstract

**Background and objectives:**

Antiglycine receptor (anti-GlyR) antibody mediates multiple immune-related diseases. This study aimed to summarize the clinical features to enhance our understanding of anti-GlyR antibody-related disease.

**Methods:**

By collecting clinical information from admitted patients positive for glycine receptor (GlyR) antibody, the clinical characteristics of a new patient positive for GlyR antibody were reported in this study. To obtain additional information regarding anti-GlyR antibody-linked illness, clinical data and findings on both newly reported instances in this study and previously published cases were merged and analyzed.

**Results:**

A new case of anti-GlyR antibody-related progressive encephalomyelitis with rigidity and myoclonus (PERM) was identified in this study. A 20-year-old man with only positive cerebrospinal fluid anti-GlyR antibody had a good prognosis with first-line immunotherapy. The literature review indicated that the common clinical manifestations of anti-GlyR antibody-related disease included PERM or stiff-person syndrome (SPS) (n = 179, 50.1%), epileptic seizure (n = 94, 26.3%), and other neurological disorders (n = 84, 24.5%). Other neurological issues included demyelination, inflammation, cerebellar ataxia and movement disorders, encephalitis, acute psychosis, cognitive impairment or dementia, celiac disease, Parkinson’s disease, neuropathic pain and allodynia, steroid-responsive deafness, hemiballism/tics, laryngeal dystonia, and generalized weakness included respiratory muscles. The group of PERM/SPS exhibited a better response to immunotherapy than others.

**Conclusions:**

The findings suggest the presence of multiple clinical phenotypes in anti-GlyR antibody-related disease. Common clinical phenotypes include PERM, SPS, epileptic seizure, and paraneoplastic disease. Patients with RERM/SPS respond well to immunotherapy.

## Introduction

The awareness of autoimmune neurologic disorders has been increasing rapidly in recent decades (1). As a result, several central nervous system (CNS) receptors and cell surface and intracellular antigens for use as pathogenic targets have been identified (2). Owing to differences in the types and distribution of antigens, the clinical manifestations also tend to vary. Common CNS autoantigens include neuronal cell-surface proteins, synaptic proteins, ion channels, and other cell-surface proteins (3).

One important inhibitory neurotransmitter is glycine (4). A proton-coupled transport mechanism accumulates glycine in synaptic vesicles, which are then released into the synaptic cleft following the depolarization of the presynaptic terminal. Pentameric GlyR, a member of the ligand-gated ion channel superfamily predominantly expressed in the brain stem, cerebellum, and spinal cord, mediates the inhibitory effect of glycine (5, 6).

Glycine receptor antibody (GlyR-Ab) was originally described in 2008 in a patient with severe progressive encephalomyelitis with rigidity and myoclonus (PERM), a variant of the stiff-person syndrome (SPS), who responded well to treatment (7). Subsequently, other PERM/SPS instances with corresponding GlyR-Ab were made public (8). GlyR-Ab individuals have also been linked to tumors and have experienced neuropathic pain, epilepsy, cerebellar ataxia, acute encephalitis with convulsions, Parkinson’s disease, and optic neuritis (9–12). As clinical cases constitute most studies conducted on GlyR-Ab so far, little is known about its epidemiology, symptomatology, and prognosis.

Initially, a case of recently identified patients who tested positive for GlyR-Ab in our investigation was reported. In addition, available data were combined with previously documented instances from the literature to determine the clinical characteristics of individuals whose blood or cerebrospinal fluid (CSF) tested positive for GlyR-Ab. Ultimately, this report aims to raise awareness of anti-GlyR antibody-related diseases.

## Methods

### Clinical features of a new patient with anti-GlyR antibody positivity

Clinical data were gathered for patients with GlyR-Ab positivity in serum and/or CSF, including clinical characteristics, laboratory findings, serum and/or CSF antibody tests, therapeutic methods, and prognosis. The modified Rankin scale (mRS) was applied to evaluate the severity of symptoms at onset and after treatment. The hospital’s medical ethics committee authorized the study, and written informed consent was obtained from the patients (2021-KY-0779).

### Literature search and data extraction

PubMed was searched for all GlyR-Ab-related publications in English till December 2023. The title/abstract and keyword combination search phrases were “GlyR or glycine receptor or glycine receptor antibodies,” “Progressive encephalopathy with rigidity and myoclonus or PERM,” “stiff-person syndrome or SPS,” and “glycine receptor encephalitis” to locate previously reported instances of anti-GlyR antibody in humans. Two expert reviewers scrutinized the whole text of the included papers, extracting and summarizing relevant material and resolving issues by consensus.

Clinical data were collected from all patients with anti-GlyR antibody-related illness, including current new participants and previously reported cases. Demographics, clinical data, laboratory results such as magnetic resonance imaging (MRI) of the brain, electroencephalogram (EEG), serum and CSF therapy, mRS scores at peak illness, and details of the last follow-up were included.

## Results

### Clinical features of the case

A novel case of anti-GlyR antibody-related autoimmune disease was identified. Comprehensive clinical features of the new case, test results, therapeutic strategy, and prognosis were reported. The admitted patient was a 22-year-old man. He exhibited prodromal symptoms, such as severe vomiting, fever, and diarrhea. His neurologic symptoms comprised involuntary jaw movements, dysphagia, involuntary tremors of the limbs, and difficulty walking. Neurological examination was unremarkable, except for bilateral Babinski sign, increased muscle tone of the extremities, both lower limbs muscle strength level 4, and difficulty in walking. The mRS score at admission was 4.

**Figure 1:**
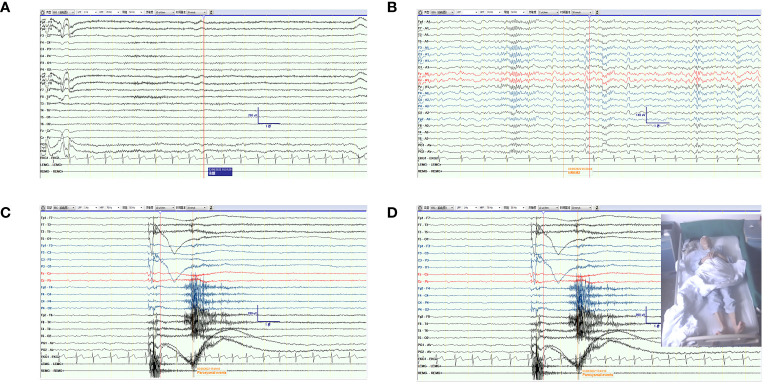
The EMG and EEG features of VEEG in the new case. **(A)** The feature of EEG and EMG in awake. **(B)** The feature of EEG and EMG in sleep. **(C, D)** The feature of EEG and EMG when the patient presented a paroxysmal event of myospasm.

The patient was examined after admission. Considering his medical history, rabies and tetanus antibody tests were performed, which turned out to be negative. MRI of the brain and spine was normal. The patient’s CSF was colorless and clear, and the pressure was 70 mmH_2_O. CSF studies revealed normal protein level (0.31 g/L, normal range: 0.15–0.45 g/L); mildly elevated white blood cell count (6/µL, normal range: 0–5/µL); and lymphocytes constituted 70%, monocytes 29%, and activated monocytes 1%. CSF bacterial culture, ink staining, and antituberculosis antibodies were negative. The serum was negative for antibodies associated with CNS demyelinating diseases, such as antibodies to aquaporin 4 (AQP-4), glial acidic fibrillary protein (GFAP), and myelin oligodendrocyte glycoprotein (MOG). Video electroencephalography (VEEG) revealed episodic involuntary upward lifting of the limbs, lasting for approximately 1 second. The patient was clearly conscious and was able to recall it later. The electromyogram (EMG) did not indicate any abnormalities. The background of the synchronized EEG did not show obvious abnormal waves or abnormal alterations in the background electrical activity. The combined findings of EMG and EEG above were indicative of myospasm. The EMG and EEG features of VEEG in the new case, including awake (A), sleep (B), myospasm (C), and (D) are presented in **Figure 1**. The serum and CSF were tested for the presence of autoimmune encephalitis antibodies using cell–based indirect immunofluorescence (Euroimmun, Lübeck, Germany) based on patient findings and clinical presentation (including anti-NMDAR, anti-LGI1, anti-CASPR2, anti-GABABR, anti-AMPRA, anti-IgLON5, anti-DPPX, anti-GAD65, anti-mGluR5, anti-GlyR5, anti-D2R, anti-MOG, and neurexin-3). The results were negative in the serum and CSF, except for anti-GlyR antibodies at a titer of 1:3.2 in the CSF. Furthermore, the results of tumor marker detection in serum were unremarkable.

The patient had an acute onset of the disease involving the brainstem, pyramidal tract, and spinal cord, and the CSF was positive for anti-GlyR antibodies. He was classified as PERM although his antibody titers were low. In previous studies, serum was more positive than CSF antibodies. A further search of the literature found no cases of positive CSF only. Therefore, the new case described in this paper is the first case positive only for CSF GlyR-Ab. The difference between CSF and serum results might be explained by the intrathecal synthesis of the GlyR-ab. The patient received first-line immunotherapy [intravenous methylprednisolone (IVMP), intravenous immunoglobulin (IVIG), plasma exchange (PE), and oral prednisone]. Initially, IVMP 1,000 mg/day was administered for 2 days, which was then halved every 3 days (total number of days on IVMP: 11). The patient’s symptoms did not improve considerably, and the mRS score remained at 4. Subsequently, he was treated with IVIG 22.5 g/day for 4 days. The mRS score improved from 4 to 3. PE was then performed four times to further alleviate the patient’s symptoms. The mRS score decreased from 3 to 1, and the serum and CSF tested negative for autoimmune encephalitis antibodies. Oral prednisone 60 mg/day was prescribed at discharge, the dose was reduced by 10 mg per week and maintained at 10 mg/day for a long time (total number of days on oral prednisone: 180). After the 6-month follow-up, the patient returned to a normal life and the mRS score was 0.

### Review of the literature

After analyzing the literature, 356 cases of anti-GlyR antibody-related illness that had already been documented were identified. These were integrated with the new patient’s data from the ongoing investigation, and **Table 1** provides a complete overview of the data.

**Table 1:** Clinical features of anti-GlyR positive patients in this new and previous literature.

	PERM/SPS	Epileptic seizure^c^	Other neurological performance^d^
Number of patients (n, %)^a^	179 (50.1%)	94 (26.3%)	84 (23.5%)
Male:Female (n, %)^b^	47:37 (56.0%:44%)	24:34 (41.4%:58.6%)	13:29 (31.0%:69.0%)
Mean age at onset (years)	47.2	32.6	40.9
Age of onset range (years)	1-89	1-79	8-89
Previous tumors (n, %)^e^	19 (10.6%)	3 (3.2%)	0
Newly discovered tumors (n, %)^f^	12 (6.7%)	0	8 (9.5%)
Abnormal MRI (n, %)	4 (2.2%)	22 (23.4%)	5 (6.0%)
Abnormal EEG (n, %)	5 (2.8%)	18 (19.2%)	8 (9.5%)
Relief symptoms after immunotherapy (n, %)	95 (53.1%)	33 (35.1%)	21 (25.0%)
Lack of improvement/Unkonwn (n, %)	84 (46.9%)	61 (64.9%)	63 (75.0%)

MRI: magnetic resonance imaging; EEG: electroencephalogram.

a Total number of 357: 1 newly detected case of PERM, 356 previous cases.

b Sex-unknown is not included.

c The forms of epileptic seizure: focal epilepsy, temporal lobe epilepsy, epileptic encephalopathy, status epilepticus, and myoclonic epilepsy, opsoclonus-myoclonus.

d Other neurological presentation: 24 demyelination/inflammatory, 24 cerebellar ataxia and movement disorders, 16 encephalitis, 7 acute psychosis, 5 cognitive impairment or dementia, 2 celiac disease, 1 Parkinson’s disease, 1 neuropathic pain and allodynia, 1 steroid responsive deafness, 1 hemiballism/tics, 1 laryngeal dystonia, 1 generalized weakness included respiratory muscles. The forms of demyelination/inflammatory disease: transverse myelitis (TM), neuromyelitis optica (NMO), isolated optic neuritis, multiple sclerosis (MS). And The forms of movement disorders: ataxic, myoclonus, motor tics, dystonia.

e A past history of tumors were reported for 19 of the 179 PERM/SPS patients, including 5 lymphoma, 4 thymoma, 2 lung cancer, 1 thymoma and lymphoma, 1 carcinoma of urinary bladder, 1 uterine cervix neoplasia, 1 tongue cancer, 1 thyroid papillary carcinoma, 1 seminoma, 1 melanoma, 1 breast cancer and thymoma. Three previous tumor patients among 86 patients in the epileptic seizure group, including 1 ovarian teratoma, 2 other preexisting malignancy. Only patients with a past history of tumors were found in the group of other neurological presentation, including 4 lung cancer, 3 breast cancer, 1 testicular seminoma.

f Tumors were mentioned for 12 of the 179 PERM/SPS patients, 6 thymoma, 2 lymphoma, 1 breast, 1 lung cancer, 1 bladder adenocarcinoma, 1 colon tubular adenoma with low-grade intraepithelial neoplasia. There were no cases of new malignancy in the group with epilepsy.

The illness associated with anti-GlyR antibodies that constituted the highest proportion was PERM/SPS (n = 179, 50.1%). Of the antibody-positive illnesses, the 94 epilepsy groups accounted for 26.3%. The forms of epileptic seizure included focal epilepsy, temporal lobe epilepsy, epileptic encephalopathy, status epilepticus, myoclonic epilepsy, and opsoclonus–myoclonus. Additionally, 23.5% of the cases were of other neurological presentations, which included 24 cases of demyelination/inflammatory disease, 24 cases of cerebellar ataxia and movement disorders, 16 cases of encephalitis, 7 cases of acute psychosis, 5 cases of cognitive impairment or dementia, 2 cases of celiac disease, 1 case of Parkinson’s disease, 1 case of neuropathic pain and allodynia, 1 case of steroid-responsive deafness, 1 case of hemiballism/tics, 1 case of laryngeal dystonia, and 1 case of generalized weakness that included the respiratory muscles. The forms of demyelination/inflammatory disease included transverse myelitis, neuromyelitis optica, isolated optic neuritis, and multiple sclerosis. The forms of movement disorders were ataxia, myoclonus, motor tics, and dystonia.

Anti-GlyR antibody-related disease can occur at any age. The minimum and maximum ages at onset were 1 and 89 years, respectively. Of the patients whose sex was known, 44% of women were in the PERM group, compared with 58.6% and 69% in the epilepsy and other neurological presentation groups, respectively. The mean age at onset in both the PERM/SPS and other neurological presentation groups was >40 years, whereas that in the epilepsy group was 32.6 years.

In the PERM/SPS group, patients with preexisting and new tumors were relatively high. A history of tumors was reported in 19 of the 179 patients with PERM/SPS, including 5 lymphomas, 4 thymomas, 2 lung cancers, 1 thymoma and lymphoma, 1 carcinoma of the urinary bladder, 1 uterine cervix neoplasia, 1 tongue cancer, 1 thyroid papillary carcinoma, 1 seminoma, 1 melanoma, and 1 breast cancer and thymoma. In addition, new tumors were mentioned for 12 of the 179 patients with PERM/SPS, including 6 thymomas, 2 lymphomas, 1 breast cancer, 1 lung cancer, 1 bladder adenocarcinoma, and 1 colon tubular adenoma with low-grade intraepithelial neoplasia. There were three patients with previous tumors among the 86 patients in the epileptic seizure group, including 1 ovarian teratoma and 2 other preexisting malignancies. However, there were no cases of new malignancy in the group with epilepsy. Only patients with a history of tumors were found in the group of other neurological presentations, including four lung cancers, three breast cancers, and one testicular seminoma. Laboratory results showed a higher proportion of MRI and EEG abnormalities in the epileptic seizure group compared with the other two groups, accounting for 23.7% and 19.4% of the total population, respectively. The corresponding proportions were 2.2% and 2.8% in the PERM/SPS group and 6.8% and 9.5% in the other neurological presentations group.

**Figure 2:**
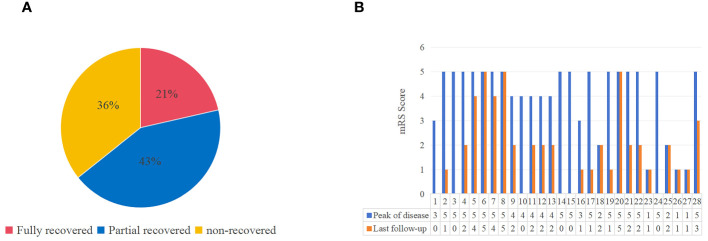
The outcome and mRS scores of patients with antiglycine receptor antibody related disease (including PERM/SPS, epileptic seizure, other neurological performance). **(A)** The outcome at the last follow-up. **(B)** Comparison of mRS scores at peak of the disease with that at the last follow-up in 28 patients with available information.

Of the known patients with applied immunotherapy, the proportion of patients in the PERM/SPS group was 53.1%, which was higher than those in the epilepsy and other neurological presentation groups (33.3% and 24.4%, respectively). Moreover, in the PERM/SPS group, according to the mRS score of the 27 patients at admission and the last follow-up, 21% (n = 6) had complete remission, 43% (n = 12) had partial remission, and 36% (n = 10) had no improvement after immunotherapy (**Figure 2**).

## Discussion

The ligand-gated, ionotropic, and inhibitory receptor GlyR of the CNS controls neurotransmission. The receptor’s heteromeric structure comprises subunits α and β (13, 14). The earliest reports of antibodies against the GlyRα1 subunit are from patients with PERM/SPS (15). In addition, GlyR antibodies have been detected in individuals with autoimmune demyelinating diseases, cerebellar ataxia, and epileptic seizures, which are symptoms of autoimmune encephalitis (16–18). One patient with GlyR-Ab positive autoimmune encephalitis who died from respiratory failure (19). The study found that patients presenting with lockjaw in SPS against GlyR-Ab had complete remission with PE, and their favorable therapeutic response clarified that GlyR-Ab directly mediated the disease (20). Moreover, an *in vivo* experiment using a zebrafish model demonstrated that GlyR-Ab directly mediated PERM/SPS (21). The importance of antibody-mediated processes in SPS and PERM is further supported by the observation that immunotherapies, such as PE, IVIG, and IVMP, improve the symptoms and simultaneously reduce serum anti-GlyR antibody levels during clinical treatment (22).

In this study, a new patient with PERM was identified. The patient was negative for serum GlyR antibody and exhibited a low CSF GlyR antibody titer of only 1:3.2. The difference between the titers in the CSF and serum could have occurred as the patient was in the initial stages of the disease and because of the intrathecal synthesis of GlyR-Ab (22–24). Previous studies have observed that positive GlyR antibody results and titers are higher in the serum than those in the CSF. The antibody was confirmed to be negative for amphiphysin and glutamic acid decarboxylase in this case, which was also the cause of PERM (25, 26). The most relevant features of PERM are myospasm, dysautonomia, brainstem dysfunction and so on, and the response to immunotherapy is variable. After first-line immunotherapy, the patient’s condition and his clinical symptoms improved significantly, which confirmed the diagnosis as GlyR-Ab-mediated PERM. In addition to the most common PERM mediated by anti-GlyR antibodies, a range of clinical syndromes can occur owing to the structural function and distribution of GlyR.

Consistent with previous studies, in this research too, anti-GlyR antibody-related diseases in addition to PERM/SPS and epilepsy were noted (10, 27–34). Moreover, demyelination/inflammation (including transverse myelitis, neuromyelitis optica, isolated optic neuritis, and multiple sclerosis) (16, 35–37), cerebellar ataxia and movement disorders, encephalitis, acute psychosis, cognitive impairment or dementia, celiac disease, Parkinson’s disease, steroid-responsive deafness, hemiballism/tics, laryngeal dystonia, and and generalized weakness included respiratory muscles were observed (12, 27, 38–41). The diversity in clinical manifestations could be attributed to GlyR-Ab acting in different areas of the brain. Knowledge of anti-GlyR antibody-related disease was further deepened by a summary of clinical phenotypes.

Autoantibodies against surface neuronal antigens have been associated with specific neurological presentations, including autoimmune encephalitis, with a variable link to neoplasia. Anti-GlyR antibody related-disease may accompany tumorigenesis (22). In addition to thymoma, lymphoma, breast cancer, lung cancer, melanoma, and seminoma reported in previous studies, bladder cancer, tongue cancer, carcinoma of the urinary bladder, uterine cervix neoplasia, tongue cancer, thyroid papillary carcinoma, and ovarian teratoma may also occur (29, 31, 32, 41–46). MRI and EEG are normal in most patients, and those with epilepsy as the clinical manifestation have a high possibility of abnormal results. As a type of autoimmune disease, anti-GlyR antibody-associated disease has a high clinical remission after the administration of immunotherapy in PERM/SPS.

This study has several limitations. First, the sample size was small, and the included studies were mostly case reports, which may be biased. In addition, follow-up information was missing for some patients, or the follow-up time was short. Hence, it was impossible to accurately evaluate the effect and long-term prognosis of immunotherapy as well as the number of patients with tumor recurrence. Further multicenter studies involving larger cohorts and longer follow-ups are required to obtain more information about the anti-GlyR antibody-related disease and enhance our understanding of clinical characteristics and prognosis.

## Conclusion

As an uncommon type of autoimmune disease, anti-GlyR antibody-related disorders mostly appear clinically as PERM, SPS, epilepsy, etc. Additional symptoms, especially unusual ones, would probably be discovered as the disease becomes increasingly recognized. Future prospective and large-cohort studies are necessary to better evaluate the clinical characteristics of this disease, given the tiny cohort of cases that have been reported so far.

## Data availability statement

The original contributions presented in the study are included in the article/supplementary material. Further inquiries can be directed to the corresponding authors.

## Ethics statement

The studies involving humans were approved by Ethics Committee of The First Affiliated Hospital of Zhengzhou University. The studies were conducted in accordance with the local legislation and institutional requirements. Written informed consent was obtained from the patients (2021-KY-0779).

## Author contributions

XW: Writing – original draft, Writing – review & editing. HZ: Conceptualization, Funding acquisition, Writing – review & editing. MS: Writing – original draft, Writing – review & editing. SF: Writing – review & editing.

## References

[1] Flanagan EP Geschwind MD Lopez-Chiriboga AS Blackburn KM Turaga S Binks S. Autoimmune encephalitis misdiagnosis in adults. JAMA Neurol. (2023) 80:30–9. doi: 10.1001/jamaneurol.2022.4251 PMC9706400 36441519

[2] Dalmau J Graus F. Diagnostic criteria for autoimmune encephalitis: utility and pitfalls for antibody-negative disease. Lancet Neurol. (2023) 22:529–40. doi: 10.1016/S1474-4422(23)00083-2 37210100

[3] Graus F Titulaer MJ Balu R Benseler S Bien CG Cellucci T. A clinical approach to diagnosis of autoimmune encephalitis. Lancet Neurol. (2016) 15:391–404. doi: 10.1016/S1474-4422(15)00401-9 26906964 PMC5066574

[4] Bowery NG Smart TG. GABA and glycine as neurotransmitters: a brief history. Br J Pharmacol. (2006) 147 Suppl 1:S109–19. doi: 10.1038/sj.bjp.0706443 PMC1760744 16402094

[5] Yu J Zhu H Lape R Greiner T Du J Lü W. Mechanism of gating and partial agonist action in the glycine receptor. Cell. (2021) 184:957–968.e21. doi: 10.1016/j.cell.2021.01.026 33567265 PMC8115384

[6] Laboute T Zucca S Holcomb M Patil DN Garza C Wheatley BA. Orphan receptor GPR158 serves as a metabotropic glycine receptor: mGlyR. Science. (2023) 379:1352–8. doi: 10.1126/science.add7150 PMC10751545 36996198

[7] Piotrowicz A Thümen A Leite MI Vincent A Moser A. A case of glycine-receptor antibody-associated encephalomyelitis with rigidity and myoclonus (PERM): clinical course, treatment and CSF findings. J Neurol. (2011) 258:2268–70. doi: 10.1007/s00415-011-6078-x 21541785

[8] Mas N Saiz A Leite MI Waters P Baron M Castaño D. Antiglycine-receptor encephalomyelitis with rigidity. J Neurol Neurosurg Psychiatry. (2011) 82:1399–401. doi: 10.1136/jnnp.2010.229104 21148607

[9] Soleimani B Board C Yu T Tracey I Irani SR Foley P. Immunotherapy-responsive neuropathic pain and allodynia in a patient with glycine receptor autoantibodies: A case report. Neurol Neuroimmunol Neuroinflamm. (2023) 10. doi: 10.1212/NXI.0000000000200160 PMC10462052 37640544

[10] Sanli E Akbayir E Kuçukali CI Baykan B Sirin NG Bebek N. Adaptive immunity cells are differentially distributed in the peripheral blood of glycine receptor antibody-positive patients with focal epilepsy of unknown cause. Epilepsy Res. (2021) 170:106542. doi: 10.1016/j.eplepsyres.2020.106542 33387801

[11] Dalakas MC. Stiff-person syndrome and GAD antibody-spectrum disorders: GABAergic neuronal excitability, immunopathogenesis and update on antibody therapies. Neurotherapeutics. (2022) 19:832–47. doi: 10.1007/s13311-022-01188-w PMC9294130 35084720

[12] Joo JY Chang HJ Woo KA Lee HS Kim HJ. Glycine receptor antibody associated stiff person syndrome with nigrostriatal dopamine loss and levodopa responsiveness. Parkinsonism Relat Disord. (2023) 111:105404. doi: 10.1016/j.parkreldis.2023.105404 37121192

[13] Rajendra S Lynch JW Schofield PR. The glycine receptor. Pharmacol Ther. (1997) 73:121–46. doi: 10.1016/S0163-7258(96)00163-5 9131721

[14] Gibbs E Klemm E Seiferth D Kumar A Ilca SL Biggin PC. Conformational transitions and allosteric modulation in a heteromeric glycine receptor. Nat Commun. (2023) 14:1363. doi: 10.1038/s41467-023-37106-7 36914669 PMC10011588

[15] Hinson SR Lopez-Chiriboga AS Bower JH Matsumoto JY Hassan A Basal E. Glycine receptor modulating antibody predicting treatable stiff-person spectrum disorders. Neurol Neuroimmunol Neuroinflamm. (2018) 5:e438. doi: 10.1212/NXI.0000000000000438 29464188 PMC5813079

[16] Woodhall M Çoban A Waters P Ekizoğlu E Kürtüncü M Shugaiv E. Glycine receptor and myelin oligodendrocyte glycoprotein antibodies in Turkish patients with neuromyelitis optica. J Neurol Sci. (2013) 335:221–3. doi: 10.1016/j.jns.2013.08.034 24045088

[17] Ariño H Gresa-Arribas N Blanco Y Martínez-Hernández E Sabater L Petit-Pedrol M. Cerebellar ataxia and glutamic acid decarboxylase antibodies: immunologic profile and long-term effect of immunotherapy. JAMA Neurol. (2014) 71:1009–16. doi: 10.1001/jamaneurol.2014.1011 PMC4841264 24934144

[18] Wuerfel E Bien CG Vincent A Woodhall M Brockmann K. Glycine receptor antibodies in a boy with focal epilepsy and episodic behavioral disorder. J Neurol Sci. (2014) 343:180–2. doi: 10.1016/j.jns.2014.05.014 24880541

[19] Reniers W Ernon L Bekelaar K. A fatal case of glycine receptor antibody-mediated autoimmune encephalitis. Acta Neurol Belg. (2021) 121:269–70. doi: 10.1007/s13760-020-01590-1 33449270

[20] Doppler K Schleyer B Geis C Grünewald B Putz E Villmann C. Lockjaw in stiff-person syndrome with autoantibodies against glycine receptors. Neurol Neuroimmunol Neuroinflamm. (2016) 3:e186. doi: 10.1212/NXI.0000000000000186 26767190 PMC4701138

[21] Rauschenberger V von Wardenburg N Schaefer N Ogino K Hirata H Lillesaar C. Glycine receptor autoantibodies impair receptor function and induce motor dysfunction. Ann Neurol. (2020) 88:544–61. doi: 10.1002/ana.25832 32588476

[22] Carvajal-González A Leite MI Waters P Woodhall M Coutinho E Balint B. Glycine receptor antibodies in PERM and related syndromes: characteristics, clinical features and outcomes. Brain. (2014) 137:2178–92. doi: 10.1093/brain/awu142 PMC4107739 24951641

[23] Crisp SJ Balint B Vincent A. Redefining progressive encephalomyelitis with rigidity and myoclonus after the discovery of antibodies to glycine receptors. Curr Opin Neurol. (2017) 30:310–6. doi: 10.1097/WCO.0000000000000450 28306573

[24] McKeon A Martinez-Hernandez E Lancaster E Matsumoto JY Harvey RJ McEvoy KM. Glycine receptor autoimmune spectrum with stiff-man syndrome phenotype. JAMA Neurol. (2013) 70:44–50. doi: 10.1001/jamaneurol.2013.574 23090334 PMC3718477

[25] Jazebi N Rodrigo S Gogia B Shawagfeh A. Anti-glutamic acid decarboxylase (GAD) positive cerebellar Ataxia with transitioning to progressive encephalomyelitis with rigidity and myoclonus (PERM), responsive to immunotherapy: A case report and review of literature. J Neuroimmunol. (2019) 332:135–7. doi: 10.1016/j.jneuroim.2019.04.003 31015081

[26] Bernardo F Rebordão L Rêgo A Machado S Passos J Costa C. Stiff person spectrum disorders: An illustrative case series of their phenotypic and antibody diversity. J Neuroimmunol. (2020) 341:577192. doi: 10.1016/j.jneuroim.2020.577192 32087460

[27] Kalampokini S Motkova I Bargiotas P Artemiadis A Zis P Hadjigeorgiou GM. A case of unusual presentation with anti-glycine receptor (GlyR) and myelin oligodentrocyte glycoprotein (MOG) antibody. Clin Park Relat Disord. (2023) 8:100195. doi: 10.1016/j.prdoa.2023.100195 37091118 PMC10119963

[28] de Freitas Dias B Fieni Toso F Slhessarenko Fraife Barreto ME de Araújo Gleizer R Dellavance A Kowacs PA. Brazilian autoimmune encephalitis network (BrAIN): antibody profile and clinical characteristics from a multicenter study. Front Immunol. (2023) 14:1256480. doi: 10.3389/fimmu.2023.1256480 37954587 PMC10634608

[29] Swayne A Warren N Prain K Gillis D O’Gorman C Tsang BK. An Australian state-based cohort study of autoimmune encephalitis cases detailing clinical presentation, investigation results, and response to therapy. Front Neurol. (2021) 12:607773. doi: 10.3389/fneur.2021.607773 33692738 PMC7937705

[30] Symonds JD Moloney TC Lang B McLellan A O’Regan ME MacLeod S. Neuronal antibody prevalence in children with seizures under 3 years: A prospective national cohort. Neurology. (2020) 95:e1590–8. doi: 10.1212/WNL.0000000000010318 32690789

[31] Piquet AL Khan M Warner JEA Wicklund MP Bennett JL Leehey MA. Novel clinical features of glycine receptor antibody syndrome: A series of 17 cases. Neurol Neuroimmunol Neuroinflamm. (2019) 6:e592. doi: 10.1212/NXI.0000000000000592 31355325 PMC6624144

[32] Matsui N Tanaka K Ishida M Yamamoto Y Matsubara Y Saika R. Prevalence, clinical profiles, and prognosis of stiff-person syndrome in a Japanese nationwide survey. Neurol Neuroimmunol Neuroinflamm. (2023) 10. doi: 10.1212/NXI.0000000000200165 PMC10519438 37739810

[33] Tekturk P Baykan B Erdag E Peach S Sezgin M Yapici Z. Investigation of neuronal auto-antibodies in children diagnosed with epileptic encephalopathy of unknown cause. Brain Dev. (2018) 40:909–17. doi: 10.1016/j.braindev.2018.06.002 29935963

[34] Vanli-Yavuz EN Erdag E Tuzun E Ekizoglu E Baysal-Kirac L Ulusoy C. Neuronal autoantibodies in mesial temporal lobe epilepsy with hippocampal sclerosis. J Neurol Neurosurg Psychiatry. (2016) 87:684–92. doi: 10.1136/jnnp-2016-313146 27151964

[35] Hacohen Y Absoud M Woodhall M Cummins C De Goede CG Hemingway C. Autoantibody biomarkers in childhood-acquired demyelinating syndromes: results from a national surveillance cohort. J Neurol Neurosurg Psychiatry. (2014) 85:456–61. doi: 10.1136/jnnp-2013-306411 24133290

[36] Martinez-Hernandez E Sepulveda M Rostásy K Höftberger R Graus F Harvey RJ. Antibodies to aquaporin 4, myelin-oligodendrocyte glycoprotein, and the glycine receptor α1 subunit in patients with isolated optic neuritis. JAMA Neurol. (2015) 72:187–93. doi: 10.1001/jamaneurol.2014.3602 PMC4836943 25506781

[37] Atmaca MM Gurses C. Status epilepticus and multiple sclerosis: A case presentation and literature review. Clin EEG Neurosci. (2018) 49:328–34. doi: 10.1177/1550059417693732 29161897

[38] Kass-Iliyya L Sarrigiannis PG Sanders DS Hadjivassiliou M. Glycine receptor antibodies and coeliac disease-related neurological dysfunction. Cerebellum Ataxias. (2021) 8:12. doi: 10.1186/s40673-021-00135-3 33941280 PMC8094486

[39] Hansen N Bartels C Stöcker W Wiltfang J Fitzner D. Impaired verbal memory recall in patients with axonal degeneration and serum glycine-receptor autoantibodies-case series. Front Psychiatry. (2021) 12:778684. doi: 10.3389/fpsyt.2021.778684 35153852 PMC8831910

[40] Lennox B Xiong W Waters P Coles A Jones PB Yeo T. The serum metabolomic profile of a distinct, inflammatory subtype of acute psychosis. Mol Psychiatry. (2022) 27:4722–30. doi: 10.1038/s41380-022-01784-4 PMC7613906 36131046

[41] Swayne A Tjoa L Broadley S Dionisio S Gillis D Jacobson L. Antiglycine receptor antibody related disease: a case series and literature review. Eur J Neurol. (2018) 25:1290–8. doi: 10.1111/ene.13721 PMC6282944 29904974

[42] Ali AH Benterud A Holmøy T Myro AZ. Progressive encephalomyelitis with rigidity and myoclonus (PERM) associated with anti-glycine receptor antibodies and urothelial carcinoma: a case report. J Med Case Rep. (2023) 17:330. doi: 10.1186/s13256-023-04059-w 37533037 PMC10399042

[43] Papantoniou M Sotiriou K. Paraneoplastic Stiff-Person syndrome with adenocarcinoma of the bladder and anti-glycine receptor antibodies. Acta Neurol Belg. (2023) 124:335–7. doi: 10.1007/s13760-023-02325-8 37405622

[44] Klein da Costa B de Oliveira Pinto P Staub L Hansel G Vanik Pinto G Porcello Schilling L. Neurological syndromes and potential triggers associated with antibodies to neuronal surface antigens. Mult Scler Relat Disord. (2023) 80:105022. doi: 10.1016/j.msard.2023.105022 37864878

[45] Yao Q Fu M Ren L Lin C Cao L. Inspiratory laryngeal stridor as the main feature of progressive encephalomyelitis with rigidity and myoclonus: a case report and literature review. BMC Neurol. (2022) 22:42. doi: 10.1186/s12883-022-02555-y 35090404 PMC8796497

[46] Nanaura H Kataoka H Kiriyama T Eura N Iwasa N Shobatake R. Spinal segmental myoclonus in both legs associated with antibodies to glycine receptors. Neurol Clin Pract. (2019) 9:176–7. doi: 10.1212/CPJ.0000000000000557 PMC6461428 31041137

